# A Pragmatic Randomized Controlled Trial of a CKD-Specific Virtual Monitoring Platform to Minimize Adverse Outcomes in High-Risk CKD Patients: A Clinical Research Protocol

**DOI:** 10.1177/20543581251359736

**Published:** 2025-10-05

**Authors:** Zahra Solati, Paul Komenda, Navdeep Tangri, Sakshi Saul, Clara Bohm, Thomas Ferguson, Drew Hager, Bryce Barr, Priyanka Mysore, Ingrid Hougen, Alejandro Meraz-Muñoz, Arsh K. Jain, Paul Tam, Claudio Rigatto

**Affiliations:** 1 Chronic Disease Innovation Centre, Seven Oaks General Hospital, Winnipeg, MB, Canada; 2 Section of Nephrology, Department of Internal Medicine, Max Rady College of Medicine, University of Manitoba, Winnipeg, Canada; 3 Department of Community Health Sciences, Max Rady College of Medicine, University of Manitoba, Winnipeg, Canada; 4 Division of Nephrology, Department of Medicine, Western University, London, ON, Canada; 5 Division of Nephrology, Scarborough General Hospital, Toronto, ON, Canada

**Keywords:** virtual monitoring, chronic kidney disease, suboptimal dialysis initiation, stage 5 CKD, digital health

## Abstract

**Background:**

The transition from advanced Chronic Kidney Disease (CKD) to dialysis is a period of heightened vulnerability for many patients. Virtual monitoring of these patients could facilitate the communication of accurate and reliable data between patients and health care providers, helping to avoid unnecessary emergency department (ED) visits and facilitate more optimal dialysis starts.

**Objective:**

To determine whether the addition of the VIEWER (Virtual Ward Incorporating Electronic Wearables) platform to usual care will lead to a reduction in ED visits and hospitalizations and lead to an increase in perceived safety of virtual care among patients and providers.

**Design:**

This study is a national, pragmatic, multicenter randomized controlled trial comparing usual care alone vs usual care plus the VIEWER virtual care platform in patients with advanced CKD. Given the nature of the intervention, patients and care providers will not be blinded; outcome assessment and statistical analysis will be blinded.

**Setting:**

Five CKD clinics in 2 Canadian provinces (Manitoba and Ontario).

**Participants:**

Patients with advanced CKD not on dialysis (eGFR <15 mL/min/1.73 m^2^, 2-year kidney failure risk >40%).

**Measurements:**

Participants randomized to the intervention group will be provided with the VIEWER platform, comprised of a wireless blood pressure (BP) monitor, weight scale, transcutaneous O_2_ sat (SpO2) monitor, wearable motion tracker, and mobile tablet running the VIEWER application. The intervention group will be trained to use the VIEWER platform to complete a daily self-assessment via the app (BP, weight, O_2_ saturation, step count) and weekly Edmonton Symptom Assessment System Revised (ESAS-r) survey. These assessments will be integrated into clinical decision-making in multidisciplinary kidney health clinics. Participants will use the VIEWER platform for 12 months (or until dialysis initiation) in addition to receiving usual care.

**Methods:**

Intention-to-treat (ITT) approach will be used to compare the primary outcome between 2 study groups. Time to primary and secondary outcomes will be assessed using univariate Cox proportional hazards models and a Kaplan-Meier analysis with a log-rank test. The primary outcome is the time to first hospital admission and/or ED visit. The control is usual care (no exposure to VIEWER platform).

**Limitations:**

Some individuals may face challenges with technology adoption, which could affect participation. Those without Internet access are limited in their ability to take part in this study.

**Conclusions:**

This study will help determine whether virtual monitoring in advanced CKD patients can reduce ED visits and hospitalization.

**Trial registration:**

Clinicaltrials.gov; identifier: NCT05726526.

## Graphical abstract

**Visual Abstract:**
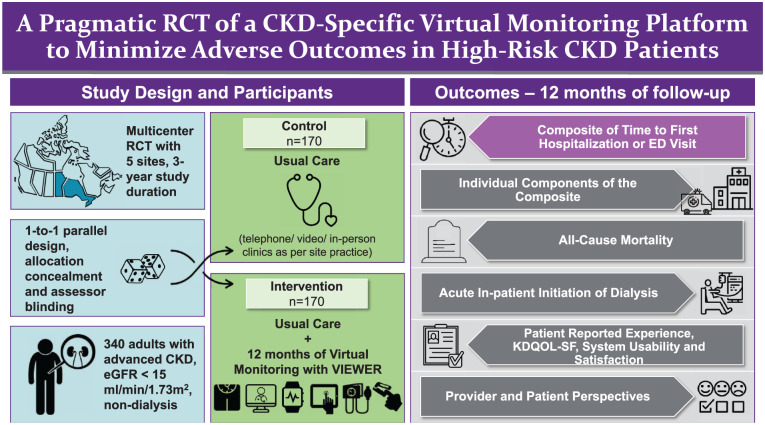

## Abrégé

**Contexte:**

Le passage à la dialyse est une période de grande vulnérabilité pour de nombreux patients atteints d’insuffisance rénale chronique (IRC) avancée. La surveillance virtuelle de ces personnes pourrait faciliter la communication de données précises et fiables à leurs prestataires de soins, ce qui pourrait aider à optimiser l’amorce de la dialyse et à limiter les visites inutiles aux urgences (URG).

**Objectif:**

Déterminer si l’ajout de la plateforme VIEWER (Virtual Ward Incorporated Electronic Wearables) aux soins habituels de dialyse entraînera une réduction du nombre de visites aux urgences et d’hospitalisations. On souhaite également déterminer si cela contribuera à renforcer le sentiment de sécurité associé aux soins virtuels chez les patients et les professionnels de la santé.

**Conception:**

Cette étude nationale, menée auprès de patients atteints d’IRC avancée, est un essai contrôlé randomisé, pragmatique et multicentrique comparant les soins habituels seuls (groupe témoin) à ces mêmes soins auxquels est ajouté un accès à la plateforme de soins virtuels VIEWER (groupe d’intervention). Compte tenu de la nature de l’intervention, la participation des patients et des prestataires de soins ne pourra être faite en aveugle; l’évaluation des résultats et l’analyse statistique seront quant à elles menées en aveugle.

**Cadre:**

Cinq cliniques d’IRC dans deux provinces canadiennes (Manitoba et Ontario).

**Sujets:**

Patients atteints d’IRC avancée non dialysés (DFGe <15 ml/min/1,73 m^2^, risque d’insuffisance rénale à 2 ans >40 %).

**Mesures:**

Les personnes randomisées dans le groupe d’intervention recevront la plateforme VIEWER, composée d’un moniteur de pression artérielle (PA) sans fil, d’un pèse-personne, d’un moniteur transcutané O2 SAT (SpO2), d’un dispositif portable de suivi des mouvements et d’une tablette exécutant l’application VIEWER. Le groupe d’intervention sera formé pour utiliser la plateforme VIEWER et effectuer une auto-évaluation quotidienne via l’application (PA, poids, saturation en O_2_, nombre de pas) ainsi qu’un sondage hebdomadaire du Edmonton Symptom Assessment System Revised (ESAS-r). Ces évaluations seront intégrées à la prise de décisions cliniques dans les cliniques multidisciplinaires de santé rénale. Les participants utiliseront la plateforme VIEWER pendant 12 mois (ou jusqu’à l’initiation de la dialyse) en plus de recevoir les soins habituels.

**Méthodologie:**

L’approche en intention de traiter (AIT) sera utilisée pour comparer le critère de jugement principal entre les deux groupes à l’étude. Le délai d’obtention des résultats primaires et secondaires sera évalué à l’aide de modèles univariés de risques proportionnels de Cox et d’une analyse Kaplan-Meier avec test logarithmique par rang. Le critère de jugement principal est le temps écoulé avant la première admission à l’hôpital et/ou la première visite aux urgences. Le groupe témoin recevra les soins habituels (sans exposition à la plateforme VIEWER).

**Limites:**

L’adoption de la technologie pourrait représenter un défi pour certaines personnes, ce qui pourrait affecter la participation. Les personnes qui n’ont pas accès à Internet sont limitées dans leur capacité à participer à cette étude.

**Conclusion:**

Cette étude aidera à déterminer si la surveillance virtuelle des patients atteints d’IRC avancée peut réduire le nombre de visites aux urgences et les hospitalisations.

**Enregistrement de l’essai:**

ClinicalTrials.gov; identifiant: NCT05726526.

## Introduction

More than 1 in 8 Canadians have chronic kidney disease (CKD), and more than 1 in 3 adults over 65 suffer from CKD and its related conditions.^1,2^ The most common causes of CKD are diabetes and hypertension, and in patients with these conditions, the prevalence may be even higher.^2^ The risk of progression to kidney failure varies depending on CKD stage (G1-G5) and degree of albuminuria (A1-A3).^3^ Patients who develop kidney failure require the initiation of renal replacement therapy, usually dialysis.

The transition from advanced CKD to the start of dialysis is a period of heightened vulnerability for many patients and is associated with a high risk of Emergency Department (ED) visits, hospitalization, and emergent or suboptimal dialysis.^4^ Multiple factors are thought to contribute to this phenomenon, including frailty, multimorbidity, unexpected declines in kidney function, or sudden appearance of uremic symptoms.^4,5^

Novel interventions designed to monitor late-stage CKD patients at home may help identify and pre-emptively treat acute worsening of clinical status (eg, volume overload, acute worsening of uremic symptoms) in these patients, reducing the number of ED visits, hospitalizations, and suboptimal dialysis in this patient population. Such strategies have proven successful in abrogating adverse clinical outcomes in other chronic diseases such as heart failure (HF).^6^

The primary objective of this trial is to determine whether the addition of the VIEWER (Virtual Ward Incorporating Electronic Wearables) monitoring platform to usual care in patients with advanced CKD (eGFR <15 mL/min/1.73 m^2^) will lead to a reduction in ED visits and/or hospitalizations.

## Methods

### Trial Design

The VIEWER study is a national, pragmatic, multicenter randomized controlled trial conducted at 5 Canadian sites comparing usual care vs usual care + the VIEWER virtual care platform in patients with advanced CKD (eGFR <15 mL/min/1.73 m^2^). Given the nature of the intervention, patients and care providers will not be blinded; outcome assessment and statistical analysis will be blinded.

### Sample Size Considerations

Based on systematic reviews of the published literature, we estimate a 39% reduction in the hazard of an outcome event (ie, hazard ratio [HR] = 0.61 for intervention).^6,7^ Further assuming an event rate in the usual care group of 11.4% per month,^7^ an expected median follow-up of 12 months per patient, a censoring rate of 3% per month, patient attrition of 10%, 2-ided alpha = 0.05, and beta = 0.10 (90% power), we will require 340 patients total (170 per arm), 68 patients/site.

### Patient Population

The trial will be conducted at 5 sites across 2 Canadian provinces:

1. Seven Oaks General Hospital (SOGH), Winnipeg, Manitoba.
2. St. Boniface Hospital (SBH), Winnipeg, Manitoba.
3. Health Sciences Centre (HSC), Winnipeg, Manitoba.
4. London Health Sciences Centre, London, Ontario.
5. Scarborough Health Network (SHN), Scarborough, Ontario.

Inclusion and exclusion criteria are presented in Table 1. The goal is to be as pragmatic as possible in order to include the majority of patients who may be eligible candidates for the trial. Potentially eligible participants that do not meet eligibility criteria can be re-screened at a later date if eligibility changes.

**Table 1.** Summary of Inclusion and Exclusion Criteria.

Inclusion criteria	Exclusion criteria
1. >18 years of age. 2. Patient or primary caregiver can read and speak English. 3. Patient or patient’s substitute decision maker is able to provide informed consent. 4. Patient or primary care giver cognitively and physically capable and willing to use the VIEWER mobile application and perform self-measurements (ie, weight, BP). 5. Have stage 5 CKD (2 measurements of eGFR <15 mL/min/1.73 m^2^); eGFR will be calculated with the CKD-EPI equation^8^ 6. Followed in a multidisciplinary CKD clinic. 7. Have >40% chance of beginning dialysis in the next 2 years based on the Kidney Failure Risk Equation (KFRE)^9^	1. Inability of self- or caregiver-assisted self-monitoring using the VIEWER platform.

### Ethical Considerations and Privacy

This protocol received approval from the Research Institute Research Ethics Boards (REB) of the University of Manitoba (HS25046 (B2021:066)), St. Boniface Hospital (RRC/2024/2152), London Health Sciences Centre (2023-123692-86164), and Scarborough Health Network Research Institute (NEP-23-011). Informed consent was obtained from all individuals who agreed to participate in the study. All study data will be entered into a secure research database (REDCap), which is passcode protected and accessible only through the secure WRHA network at the University of Manitoba. Only the research team will have access to patient data, ensuring the privacy and security of all participants.

### Study Procedures

**Figure 1.**
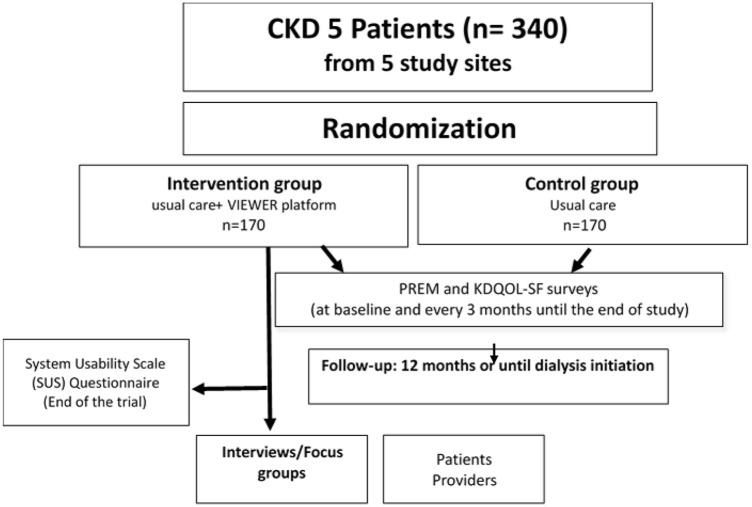
Study flow: 340 eligible participants will be randomly assigned to either the intervention group (receiving usual care along with the VIEWER virtual care platform) or the control group (receiving only usual care). Participants will be followed for 1 year or until dialysis initiation. Participants in the intervention group will receive a wireless BP cuff, weight scale, transcutaneous O_2_ saturation (SpO_2_) monitor, wearable motion tracker, and a mobile tablet with the VIEWER application. They will be trained to use the VIEWER platform while continuing to receive usual care. Participants in the control group will receive usual care without additional devices or platforms. All participants will complete the UK Kidney PREM score (adapted for CKD) and the Health-related Quality of Life using KDQOL-SF via REDCap or paper forms at baseline, 3, 6, 9, and 12 months (or every 3 months thereafter until dialysis initiation). At the end of the study, virtual interviews or focus groups will be conducted with a subset of participants from the intervention group and health care providers.

Study flow is presented in Figure 1.

### Recruitment

A research coordinator will work with renal clinic staff (ie, nurse, clinical clerk or physician) at each site to identify potential participants. Patients attending clinic will be asked by a member of their care team about whether they might be interested in the VIEWER study. A brief script describing the study in lay terms will be provided to the care team to facilitate this. If the patient agrees, the research coordinator will approach them and provide more detailed information on the trial and answer any questions. Patients will also have the option of meeting virtually with the study coordinator at a more convenient time if they prefer.

### Informed Consent

During the informed consent process, whether in-person or virtual, the patient will be provided ample time for questions and reflection, including the option to contact the research coordinator at a later date with further questions. Written informed consent will be obtained by means of the participant’s dated signature and the dated signature of the study coordinator. Patients will have the option of either virtual (via Redcap portal) or paper consent. During the consent visit, upon signing of the consent form, the study coordinator will review inclusion/exclusion criteria with the participants to ensure eligibility based on the screening form. Participant address will be collected during this meeting should they opt for study kit delivery by courier/delivery service. A copy of the signed consent form will be made available to the participant.

### Randomization

Randomization will be performed centrally within the REDCap database using randomization macros in statistical software (SAS 9.4). Patients will be randomized 1:1 to control vs VIEWER groups, stratified by site with block sizes of 8 to accommodate stratification.

### Baseline Assessment

Baseline assessment will take place within 14 days of enrollment either virtually or in-person. During the baseline visit, the research coordinator will provide general instruction on how to complete the UK Kidney PREM and the KDQOL-SF via a virtual link. Baseline data collected will include sex, gender, race, age, eGFR, kidney failure risk equation (KFRE) score, patient-reported outcome measures (PROMs) (measured by KDQOL-SF questionnaire) and patient-reported experience measures (PREMs). Demographic characteristics, eGFR, and KFRE will be collected from patient charts/electronic medical record (EMR) and entered into REDCap; KDQOL-SF and PREM questionnaires will be completed by patients via REDCap.

### Intervention Group

Participants in the intervention arm will receive training on how to use the VIEWER software and associated devices during a training session. Participants will be able to contact the study team with follow-up questions at any time during workday hours, Monday to Friday via text, phone call, and video link.

### VIEWER Description

The VIEWER is a CKD-specific virtual care platform that integrates data from a wireless blood pressure (BP) cuff, weight scale, transcutaneous O_2_ sat monitor (SpO_2_), and wearable motion tracker. The patient-facing component of VIEWER (patient portal) is a custom application based on a mobile tablet that guides patients through a daily self-assessment routine using the connected devices. The BP measurements, weight and oxygenation (as surrogates of volume status), and step counts (as a surrogate of functional status) provide semi-continuous longitudinal data on patient physical status. In addition, patients are prompted to fill out a weekly symptom survey (Edmonton Symptom Assessment Score-revised; ESAS-r), a validated instrument of kidney failure symptoms.^10^ Patient data and ESAS scores are automatically uploaded to fully PHIA/HIPPA-compliant servers, where they are made available to the patients’ care team through a secure, web-based provider portal. Provider notifications (flags) are generated for out-of-range values (ie, BP, weights for volume management) and a secure messaging component allows for direct patient-provider communication. The VIEWER platform components are presented in Table 2.

**Table 2.** VIEWER Platform Components.

VIEWER platform components
The VIEWER virtual care platform used in this study will include the following components: 1. Mobile tablet loaded with the custom VIEWER mobile application that will act as a portal for patients to upload clinical data (from connected devices) and access data trends and secure text/video communication (between patient/provider). 2. Web-based provider portal accessible through any Internet browser via a secure login. 3. Bluetooth-enabled home weight scale, BP cuff, O_2_ saturation monitor, and wearable motion tracker. 4. Secure text and video messaging between patients and providers (nurses in each clinic for management of the virtual platform). 5. Secure data transfer over the Internet. 6. Option to enable push notifications as a reminder to perform daily assessments.

Participants will be trained to use the VIEWER devices and app by a member of the research team via video conferencing call with an in-clinic option available if needed. On the provider side, assigned nurses at each site will be trained as “superusers” of the provider portal. These users will check the VIEWER platform daily and respond as needed (Monday to Friday) for flags and patient messages and will respond or communicate as needed to the health care team as per local clinic standard operating procedures. Trends in measurements will be reviewed weekly with the most responsible physician. During routine clinic visits, trends in all objective and subjective measures will be made available to inform clinical decision-making. Consistent with our pragmatic design, we will not be prescriptive in how monitoring data are interpreted; rather, we ask that care teams regularly review this enriched data stream and incorporate that information in the context of their own protocols and clinical judgment.

In addition to using the VIEWER virtual care platform, patients in the intervention group will continue to see their multidisciplinary CKD care teams as per local standard of care, either virtually or in-person.

### Control Group

Patients randomized to the control group will see their multidisciplinary CKD care teams as per usual care, either virtually or in-person.

### Non-Study Treatments

Responsibility for the management of participating patients rests with their own clinicians, who are free to initiate, alter, or cease any treatment as clinically appropriate. Specifically, management of hypertension, volume status, acidosis, potassium, anemia, bone mineral metabolism, cardiovascular risk reduction, infection, dialysis modality planning and initiation, kidney transplant referral, and advanced care planning will be left to the discretion of the patients’ clinical care team.

### Follow-up Visits

Patient-Reported Outcome Measures (using KDQOL-SF) and Experience Measures (PREM) will be measured at baseline, 3, 6, 9, and 12 months (or at baseline and subsequently every 3 months until the initiation of the dialysis). The validated tools used will be the UK Kidney PREM (adapted to CKD), which measures several patient experience domains relevant to CKD, and the KDQOL-SF, which measures health-related quality of life. Each of these surveys takes about 15 to 20 minutes to complete.

Usability, acceptability, and patient perspective on the intervention will be assessed at the end of the trial (12 months or at the time of dialysis initiation) using the System Usability Scale (SUS) survey, which will be sent via REDCap link.

Finally, the occurrence of primary and secondary outcomes (defined below) will be assessed at all follow-up visits.

### Exit Focus Groups/Interviews

Participants in the intervention group will be asked to participate in a virtual exit focus group/interview at the end of the study (ie, at 12-month follow-up or upon dialysis initiation). Informed consent will be obtained to record and transcribe the focus groups/interviews for thematic analysis. The purpose of these focus groups/interviews will be to describe the subjective patient experiences of using VIEWER. The focus groups/interviews will be semi-structured, and interviewers will be provided a list of topic areas germane to the patient experience that should be discussed during the focus group/interview.

## Outcomes

### Primary Outcome

Our composite primary outcome will be time to first hospitalization or first ED visit.

### Secondary Outcomes

1. Individual components of the composite outcome.
2. All-cause mortality.
3. Acute inpatient initiation of dialysis.
4. UK Kidney PREM score (adapted to CKD).
5. Kidney Disease Quality of Life-Short Form (KDQOL-SF) score.
6. System Usability Scale (SUS).
7. Frequency of formal clinic visits.
8. Adherence.
9. Qualitative outcomes: provider and patient perspectives (qualitative).

## Statistical Methods

### Primary Analysis

Our primary analysis will take an intention-to-treat (ITT) approach. The primary outcome (time to first hospitalization or ED visit, censoring at dialysis or death) will be compared between groups using a Kaplan-Meier analysis with a log-rank test.

### Secondary Outcomes and Sensitivity Analyses

The same Kaplan-Meier survival curve approach will be used for the secondary outcomes of all-cause mortality, ED visits, hospitalization, and acute inpatient dialysis initiation. Change in overall KDQOL, UK Kidney PREM, and SUS scores will be assessed with a 2-sided t-test or Wilcoxon Rank Sum test as distributionally appropriate and interpreted relative to their minimal important differences. Sensitivity analyses will include (1) negative binomial regression and mixed linear models to account for repeated events; (2) multivariate Cox regression to adjust for any potential imbalances in prognostic covariates; and (3) an Andersen-Gill model to adjust for repeated events in the context of a time-to-event analysis.^11^ All outcomes will be assessed at a 2-sided alpha = 0.05. Subgroup analyses will be prespecified and limited to (1) eGFR greater or less than 10 mL/min, (2) sex and gender (described below), and (3) diabetes. A per-protocol analysis will be performed specifically and only to estimate the potential impact of patient non-adherence.

### Qualitative Analysis

For the analysis and interpretation of the exit focus groups/interviews, we will use a qualitative descriptive methodology.^12^ Thematic analysis will be performed with the assistance of NVIVO. Thematic codes will be categorized and analyzed by 2 independent researchers using summative content analysis, incorporating both inductive and deductive approaches in the analysis.^13^

### Economic Assessment

We will use event rates and direct costs derived from the trial to re-calibrate our previously published Markov cost-effectiveness model in determining the cost utility of this intervention.^14^

### Sex and Gender Differences

We will collect information on both sex and gender at enrollment. We will perform a prespecified subgroup analysis given known differences in CKD progression by sex. We will also examine intervention uptake and adherence by gender in a sensitivity analysis, and the primary and secondary outcomes will be adjusted for gender if there are baseline imbalances between arms.

## Discussion

We designed a pragmatic clinical trial to investigate the impact of incorporating a virtual monitoring platform (VIEWER) into standard care for high-risk CKD patients. Our hypothesis is that the addition of VIEWER to usual care will result in a decrease in the risk of an ED visit or hospitalization compared with usual care alone.

Patients with advanced CKD face a vulnerable period when transitioning to dialysis which is reflected in high rates of ED visits before dialysis initiation.^4^ In a population-level retrospective cohort study in Manitoba, Canada, Komenda et al^4^ found that in a 7-day period before dialysis initiation, new dialysis patients were 9-fold more likely than prevalent dialysis patients, and 20-fold more likely than the general population to visit the ED. Vulnerability during this time is partly attributable to risk inherent in advanced CKD population such as age, frailty, and multiple comorbidities.^4^ Precipitating causes of ED visits often include fluid overload, hyperkalemia, bacteremia, and accelerated decline in kidney function.^4^ These precipitating events may be detectable by preceding changes in BP or weight and decreases in oxygen saturation and physical activity.^15-18^ Monitoring of these variables in advanced HF appears to improve patient outcome in health failure populations.^6^ Whether a similar strategy can prevent unplanned ED visits or hospitalizations in an advanced CKD population is unknown.

Uminski et al^6^ conducted a systematic review and meta-analysis evaluating the effects of post-discharge virtual wards on hospital readmissions compared to usual care. They found that when implemented for HF patients, multidisciplinary virtual monitoring (of weight, vital signs, and symptoms) can lower the risk of all-cause mortality and heart-failure-related hospital readmissions.^6^ In addition, Bamforth et al^19^ examined post-discharge interventions and their impact on reducing the likelihood of death and hospital readmissions in patients with HF, chronic obstructive pulmonary disease (COPD), and CKD. The authors found that post-discharge interventions decreased mortality and hospital admissions in HF and in COPD patients, but there was a gap in the research on the impact of these programs in patients with CKD.^19^ Both advanced CKD and HF share a significant number of similarities and exhibit symptoms which can be identified early and managed remotely, making virtual monitoring a promising preventative tool.^6^ As such, virtual monitoring in advanced CKD patients has the potential to improve health outcomes with a monitoring program that is specifically tailored to CKD.

The VIEWER is a disease-specific intervention which is looking at monitoring parameters relevant to the care of CKD patients who are approaching kidney failure and need to start dialysis. The VIEWER was created by a team of nephrology clinicians, data scientists, patient partners, and information technology collaborators. Significant effort went into making this platform accessible and easy to use.

The VIEWER provides daily data on BP, weight, blood oxygen saturation levels (SpO_2_), and physical activity levels. We pilot tested VIEWER in 36 CKD patients (80% with diabetes, average age 74, 46% female) to assess usability, acceptability, and adherence, and gather patient feedback.^20^ Results showed high usability (90/100 on SUS scale) and acceptable adherence with daily self-assessments (78%) (18).^20^ Even though adherence decreased at 3 months, >80% of participants continued to use the kit at least twice weekly even at 3 months.^20^ In total, 70% of participants asked if they could keep using the kits after the study, suggesting high acceptability.^20^

The VIEWER, a tablet-based virtual care platform for CKD patients, has the potential to manage CKD and reduce the likelihood of adverse outcomes such as ED visits and hospitalization. The proposed trial will rigorously test whether this platform deployed in realistic clinical settings can improve important patient outcomes.

## Strengths

Our study has many strengths. We are addressing an important impactful problem for patients with advanced CKD in a rigorous scientific design (RCT). This design will minimize bias and strengthen the internal validity of the results. Inclusion of multiple centers with diverse patient populations will enhance the external validity of our study. In this regard, we have included both academic, tertiary care referral centers, as well as community practices in order to be more broadly representative of clinical practice settings in Canada. The trial will be powered to detect a meaningful change in a clinically meaningful outcome for patients. Our intervention has been designed specifically for patients with CKD. Our previous published work has taught us that this disease-specific focus in the context of a telemonitoring intervention is important.

## Limitations

Our trial has some limitations. Patients with CKD tend to be older, of lower socioeconomic status, and often have limited health and digital literacy skills.^1^,^21-23^ This could influence their engagement in this study. Previous data also showed that many individuals from remote areas encounter difficulties in both accessing and utilizing broadband Internet services.^24^ The barriers are higher in Indigenous communities that have higher rates of CKD compared to the general population.^25^ The restricted Internet access within this population could impact their involvement in this study.

## Conclusion

In summary, optimized virtual care models could improve care of patients with complex chronic disease such as CKD patients. The VIEWER is a user-friendly, highly feasible platform designed by our multidisciplinary team to enhance virtual clinical care for CKD patients and reduce adverse, burdensome, and costly outcomes such as ED visits, hospitalizations, and acute inpatient dialysis initiation. Our findings could potentially influence CKD management in Canada and internationally.
